# Nicotinamide adenine dinucleotide suppresses epileptogenesis at an early stage

**DOI:** 10.1038/s41598-017-07343-0

**Published:** 2017-08-04

**Authors:** Juan Liu, Beimeng Yang, Pei Zhou, Yingying Kong, Weiwei Hu, Geng Zhu, Weihai Ying, Weidong Li, Yun Wang, Shengtian Li

**Affiliations:** 1 Key laboratory for the Genetics of Developmental and Neuropsychiatric Disorders (Ministry of Education), Bio-X Institutes, Shanghai Key Laboratory of Psychotic Disorders, Institute of Social Cognitive and Behavioral Sciences, and Brain Science and Technology Research Center, Shanghai Jiao Tong University, 800 Dongchuan Road, Shanghai, 200240 China; 2 Institutes of Brain Science, State Key Laboratory for Medical Neurobiology, Collaborative Innovation Center for Brain Science, Fudan University, Shanghai, 200032 China; 3 School of Bimedical Engineering and Med-X Research Institute, Shanghai Jiao Tong University, Shanghai, 200030 China

## Abstract

The pathophysiologic mechanisms of epileptogenesis are poorly understood, and no effective therapy exists for suppressing epileptogenesis. Numerous reports have shown that nicotinamide adenine dinucleotide (NAD^+^) has neuroprotective effects, suggesting its potential use for treating epileptogenesis. Here we evaluated the effects of NAD^+^ on epileptogenesis and the mechanisms underlying these effects. In pilocarpine-induced status epilepticus (SE) model mice, NAD^+^ was injected three times within 24.5 h after SE. NAD^+^ intervention significantly reduced the incidence of spontaneous recurrent seizure (SRS) and abnormal electroencephalogram (EEG) activity, rescued contextual fear memory formation, reduced neuronal loss in the CA1 region of the hippocampus at SRS stage. Furthermore, exogenous supply of NAD^+^ distinctly reversed the seizure-induced depletion of endogenous NAD^+^, reduced neuronal apoptosis in the CA1 region of the hippocampus, and reversed the augmented Acp53/p53 ratio at the early stage of epileptogenesis. Our findings demonstrated that early-stage intervention with NAD^+^ prevents epileptogenesis in pilocarpine-induced SE mice by suppressing neuronal apoptosis.

## Introduction

Epilepsy is one of the most common neurological disorders, affecting at least 50 million people worldwide. Seizures, which are caused by the abnormal synchronised electrical overactivity of a group of cerebral neurons, can be convulsive or nonconvulsive episodes^1–3^. Currently, the majority of antiepileptic drugs simply alleviate seizure symptoms but do not target the underlying disease-promoting mechanisms, namely epileptogenesis. The time window of epileptogenesis starts from the occurrence of brain injuries to the development of spontaneous recurrent seizures^4^. Thus, identifying effective antiepileptogenic interventions is imperative. To date, several factors related to the development of epileptogenesis have been reported, including apoptosis, inflammatory factors, activation of the mammalian target of rapamycin pathway, glial activation, brain–blood barrier (BBB) breakdown, and oxidative stress injury^5, 6^.

Nicotinamide adenine dinucleotide (NAD^+^) is a coenzyme found in all living cells and exists in two forms, the oxidised and reduced forms of NAD^+^ and NADH, respectively. NAD^+^ has essential roles in metabolism; it acts as a coenzyme in redox reactions, as a donor of adenosine diphosphate (ADP)-ribose moieties in ADP-ribosylation reactions, as a precursor of the second messenger molecule of cyclic ADP-ribose, and as a substrate for bacterial DNA ligases and a group of enzymes called sirtuins, which use NAD^+^ to remove acetyl groups from proteins^7–11^. From 1995 to 1997, several studies have found that increased brain NAD^+^ levels prevent neuronal apoptosis^12–14^. NAD^+^ has since been studied for its potential use in the therapy of neurodegenerative diseases such as Alzheimer disease and Parkinson disease^15^. Because the pathogenesis of epileptogenesis involves neuronal apoptosis^5, 6^, the antiapoptotic effects of NAD^+^ suggest that it can be applied as an intervention for epileptogenesis. Recent studies have found that endogenous NAD^+^ is significantly depleted in the acquired epilepsy neuronal cell model caused by Mg^2+^-free incubation and that the NADH level is decreased in the hippocampus of pilocarpine-induced epilepsy model mice^16, 17^, suggesting the correlation of NAD^+^ with the pathological process of epilepsy. On the basis of this assumption, in this study, we evaluated the effects of intraperitoneal (i.p.) injection of NAD^+^ on epileptogenesis in pilocarpine-induced status epilepticus (SE) model mice. The results demonstrated that early-stage intervention with NAD^+^ after SE prevented the acetylation of p53 and the apoptosis of hippocampal neurons, thereby lowering the incidence of seizures, abnormal electroencephalogram (EEG) activity, hippocampal neuronal loss, and fear memory impairment at the spontaneous recurrent seizure (SRS) stage in the SE model mice. Our results provide the first evidence that early-stage injection of NAD^+^ after brain injury can be an effective intervention for epileptogenesis.

## Results

### NAD^+^ Injections Reversed NAD^+^ Depletion in SE Model Mice at Early-Stage Epileptogenesis

**Figure 1:**
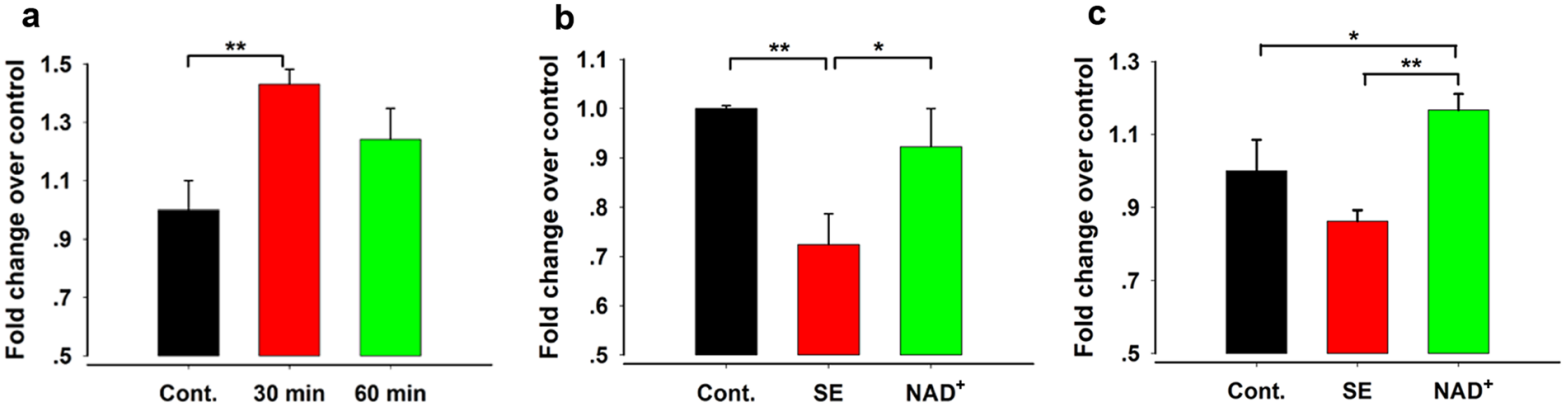
NAD^+^ injections reversed NAD^+^ depletion in SE model mice. (**a**) Exogenous supply of NAD^+^ led to significant augmentation of NAD^+^ in the hippocampus at 30 min after NAD^+^ injection (Cont., N = 5; 30 min, N = 6; 60 min, N = 5). (**b**,**c**) Changes in the NAD^+^ level in the hippocampus of control, SE model mice, and NAD^+^-treated SE mice at 24.5 h (Cont., N = 5; SE, N = 6; NAD^+^, N = 6) and 1 h (Cont., N = 4; SE, N = 6; NAD^+^, N = 6) after SE. Cont., SE, and NAD^+^ represent the control, SE model mice, and NAD^+^-treated SE mice, respectively. Data are represented as mean ± SEM. *P < 0.05, **P < 0.01, ***P < 0.001.

To confirm whether NAD^+^ penetrates the BBB, we harvested the hippocampus and measured the NAD^+^ level at 30 min and 60 min after NAD^+^ injection in normal male C57BL/6 mice. As shown in Fig. 1a, compared with control mice (1.00 ± 0.10, N = 5), the NAD^+^ level in the hippocampus was significantly high at 30 min after the i.p. injection of NAD^+^ (100 mg/kg, i.p.; 1.43 ± 0.05, N = 6) and was still higher at 60 min after the injection (1.24 ± 0.11, N = 5) (one-way ANOVA, F(2, 13) = 6.513, *P* = 0.011; LSD post hoc, 30 min vs control, *P* = 0.003; 60 min vs control, *P* = 0.075). Moreover, we assessed and compared the NAD^+^ level at 24.5 h after SE (30 min after the third NAD^+^ injection) among control (1 ± 0.01, N = 5), SE model mice, and NAD^+^-treated SE model mice. As shown in Fig. 1b, the NAD^+^ level was significantly decreased in SE model mice (0.72 ± 0.06, N = 6), and NAD^+^ treatment (0.92 ± 0.08, N = 6) distinctly prevented this reduction (one-way ANOVA, F(2,14) = 5.329, *P* = 0.019; LSD post hoc, SE vs control, *P* = 0.008; NAD^+^ vs SE, *P* = 0.034). To determine when the NAD^+^ level begins to decrease after SE, we tested the NAD^+^ level at 1 h after the SE (Fig. 1c). The NAD^+^ level was slightly decreased at 1 h after SE (control mice, 1 ± 0.09, N = 4; SE mice, 0.86 ± 0.03, N = 6), and NAD^+^-treated SE mice exhibited higher NAD^+^ levels than SE model mice at 1 h after SE (1.17 ± 0.04, N = 6; one-way ANOVA, F(2,13) = 10.545, *P* = 0.002; LSD post hoc, NAD^+^ vs SE, *P* = 0.001). These data indicate that exogenous NAD^+^ injection reversed the depletion of endogenous NAD^+^ resulting from SE at early-stage epileptogenesis.

Furthermore, considering the essential functions of NAD^+^ in the balance of antioxidation and oxidative stress, energy metabolism, etc., the therapeutic potential of reduced NADH and oxidised nicotinamide adenine dinucleotide phosphate (NADP^+^) has also been reported^7^. In this study, biochemical assay kits were used to measure the NADH and NADP^+^ levels in the hippocampus at 24.5 h after SE. We found that both NADH (control group, 1 ± 0.079, N = 4; SE, 0.63 ± 0.05, N = 4; Student’s *t*-test, SE vs control, *P* < 0.01) and NADP^+^ (control group, 1 ± 0.027, N = 4; SE, 0.66 ± 0.02, N = 4; Student’s *t*-test, SE vs control, *P* < 0.001) were significantly decreased in SE mice, providing the vital insight that exogenous supply of NADH and NADP^+^ at early-stage epileptogenesis may exert therapeutic effects.

### Early-Stage Injection of NAD^+^ Attenuated the Incidence of Seizures in SE Model Mice at SRS Stage

**Figure 2:**
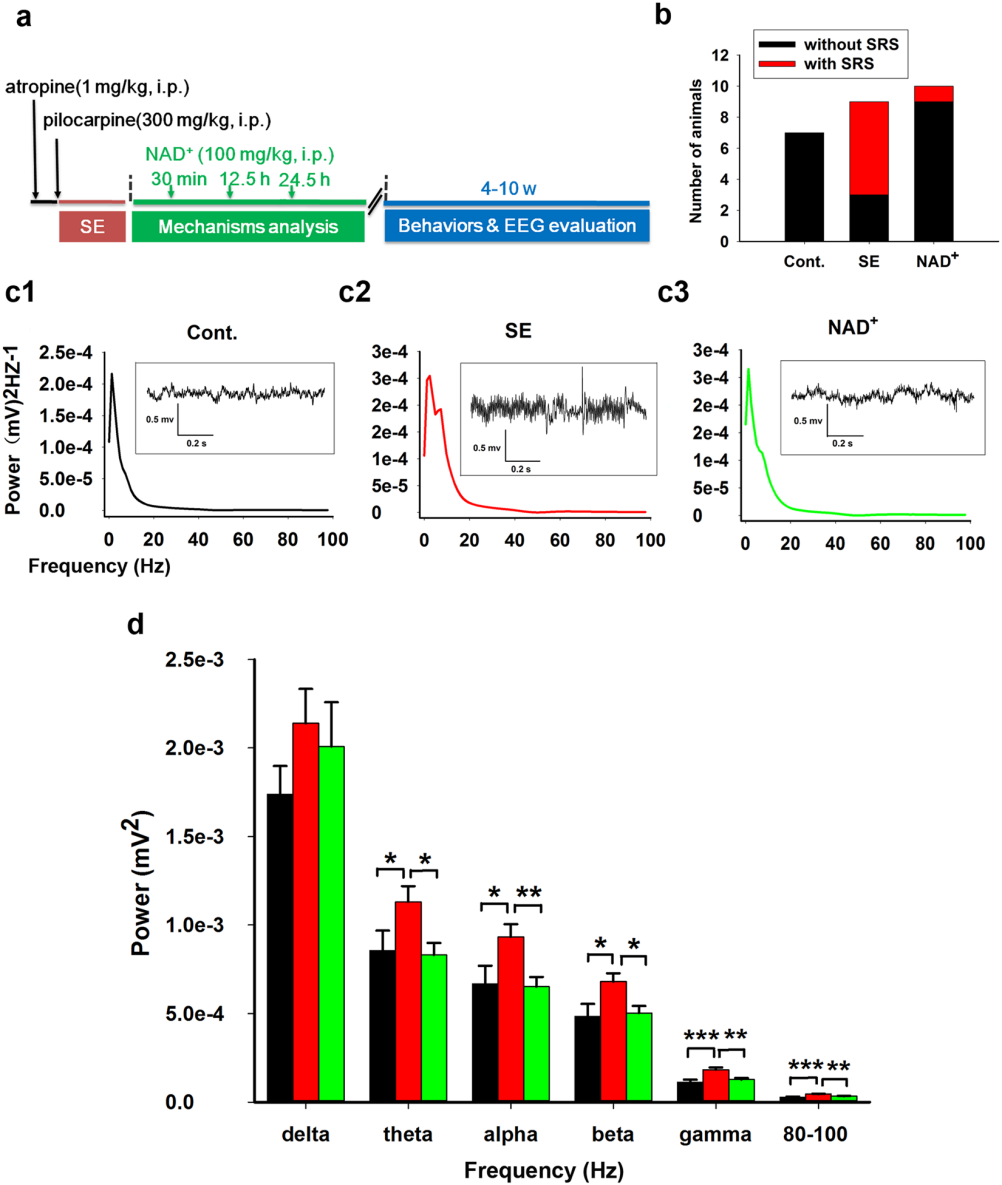
Early-stage intervention with NAD^+^ attenuated the incidence of seizures and abnormal EEG activity in SE model mice at SRS stage. (**a**) Experimental design and timeline. NAD^+^ was i.p. injected three times at 30 min, 12.5 h, and 24.5 h after SE. The incidence of spontaneous seizures and abnormal EEG activity at the SRS stage were evaluated. (**b**) NAD^+^ injection significantly reduced seizure occurrence (Cont., N = 7; SE, N = 9; NAD^+^, N = 10). The ratio of mice with spontaneous seizures to the total number of mice is shown in the bars. Power spectral values of EEG at 0–100 Hz were analysed. Power spectrum analysis of 12-h EEG from control (c1, N = 9, n = 19), SE (c2, N = 8, n = 28), and NAD^+^-treated SE group (c3, N = 10, n = 30) was performed. N refers to the number of mice and n indicates the number of days of EEG activity recording. Inset boxes show the representative EEG. (**d**) The statistical results of power values. Black, red, and green bars represent the control, SE model mice, and NAD^+^-treated SE mice, respectively. *P < 0.05, **P < 0.01, ***P < 0.001.

Considering that the occurrence of seizures at the SRS stage is a pathological consequence of pilocarpine injection, behavioural videos were analysed during the 4–10 weeks after SE. Only the seizures graded as Racine’s stage 4 or higher were analysed. As shown in Fig. 2b, pilocarpine-induced SE mice developed a constant incidence of seizures, whereas control mice did not develop any seizures. Spontaneous seizures were observed in 6 of 9 (67%) SE model mice and in 1 of 10 (10%) NAD^+^-treated model mice. These results indicated that early-stage injection of NAD^+^ significantly inhibited pilocarpine-induced epileptogenesis (Fig. 2b, Chi-square test, SE vs control, *P* = 0.011; NAD^+^ vs SE, *P* = 0.02). The mean frequency and duration of seizures were 0.50 ± 0.12 events per day and 14 ± 3.9 s in SE mice and 0.67 events per day and 6.5 s in NAD^+^-treated SE mice. The results described in this section provide the strongest evidence that NAD^+^ injection reduced the incidence of seizures in SE model mice at the SRS stage.

### Early-Stage Intervention With NAD^+^ Reversed Abnormal EEG Activity in SE Model Mice at SRS Stage

Along with behavioural video monitoring at the SRS stage, EEG activity was recorded. We conducted power spectral analysis (Fig. 2c1–3) and calculated the power values (areas under curves). Compared with control mice (N = 9, n = 19, N refers to the number of mice and n indicates the number of days of EEG activity recording), SE model mice (N = 8, n = 28) showed significantly higher EEG power values in the theta rhythm (4–7.5 Hz), alpha rhythm (8–12 Hz), beta rhythm (13–25 Hz), gamma rhythm (40–80 Hz), and 80–100 Hz frequency band, but not in the delta rhythm ( < 3.5 Hz) (Fig. 2d, one-way ANOVA, F(2, 74) = 3.038, *P* = 0.054; LSD post hoc, for SE vs control: theta, *P* = 0.039; alpha, *P* = 0.017; beta, *P* = 0.012; gamma, *P* < 0.001; 80–100 Hz, *P* < 0.001). NAD^+^ treatment (N = 10, n = 30) significantly reduced the abnormal EEG power of these bands (LSD post hoc, NAD^+^ vs SE: theta, *P* = 0.011; alpha, *P* = 0.005; beta, *P* = 0.01; gamma, *P* = 0.001; 80–100 Hz, *P* = 0.001). We also calculated the EEG power values at 100–200 Hz and 200–500 Hz and found that NAD^+^ injection did not rescue abnormal EEG activity at these frequencies among the groups (data not shown). The results indicate that early-stage intervention with NAD^+^ reversed abnormal EEG activity in SE model mice at the SRS stage.

**Figure 3:**
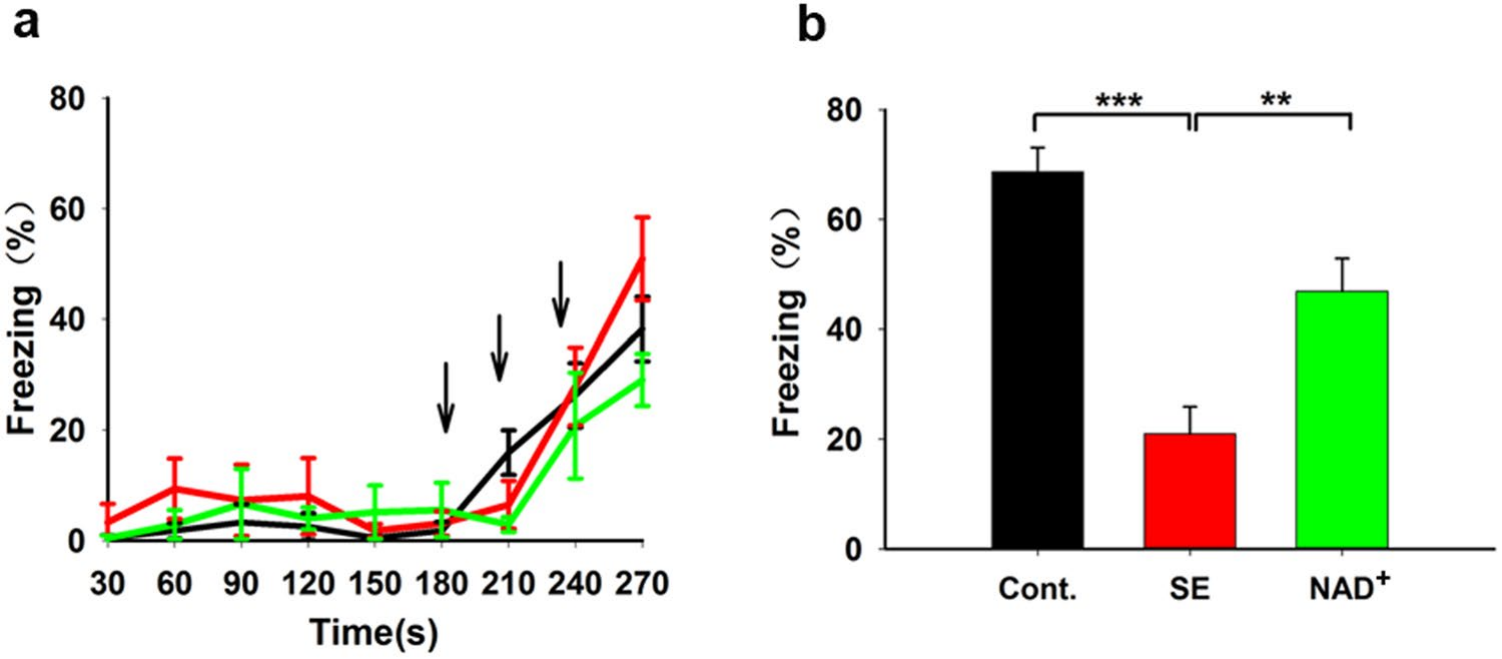
Early-stage intervention with NAD^+^ suppressed contextual memory impairment of SE model mice at SRS stage. Although the freezing response to the electrical foot shocks was not changed by treatment with either pilocarpine or pilocarpine plus NAD^+^ (**a**), the contextual fear memory was impaired in SE mice but was significantly restored in NAD^+^-treated SE mice (**b**). Black, red, and green lines represent the control (N = 11), SE model mice (N = 12), and NAD^+^-treated SE mice (N = 12), respectively. The black arrows refer to electrical foot shock. Cont., SE, and NAD^+^ represent the control, SE model mice, and NAD^+^-treated SE mice, respectively. Data are represented as mean ± SEM. *P < 0.05, **P < 0.01, ***P < 0.001.

### Early-Stage Injection of NAD^+^ Restored Contextual Fear Memory Impairment in SE Model Mice at SRS Stage

We previously found that pilocarpine injection causes fear memory deficiency^18^. To evaluate the effect of NAD^+^ on this memory deficit, we performed contextual fear conditioning tests at 7 weeks after SE. No significant difference was observed in the freezing response to the electrical foot shocks among the three groups (Fig. 3a). After twenty-four hours, they were placed in the chamber again, and pilocarpine-induced SE mice showed significantly lower freezing times than control mice, which exhibited serious contextual fear memory deficit (Fig. 3b, control mice, 68.7% ± 4.4%, N = 11; SE mice, 21% ± 4.9%, N = 12; one-way ANOVA, F(2, 32) = 20.72, *P* < 0.001; LSD post hoc, SE vs control, *P* < 0.001). In addition, compared with SE model mice, NAD^+^ treatment significantly restored the freezing time, indicating the therapeutic effect of NAD^+^ on contextual fear memory impairment in SE model mice (Fig. 3b, NAD^+^, 46.9% ± 6%, N = 12; LSD post hoc, NAD^+^ vs SE, *P* = 0.001). These results demonstrate that early-stage intervention with NAD^+^ improved contextual fear memory impairment in SE model mice at the SRS stage.

### Early-Stage Intervention With NAD^+^ Reduced Neuronal Loss in CA1 of Hippocampus in SE Model Mice at SRS Stage

**Figure 4:**
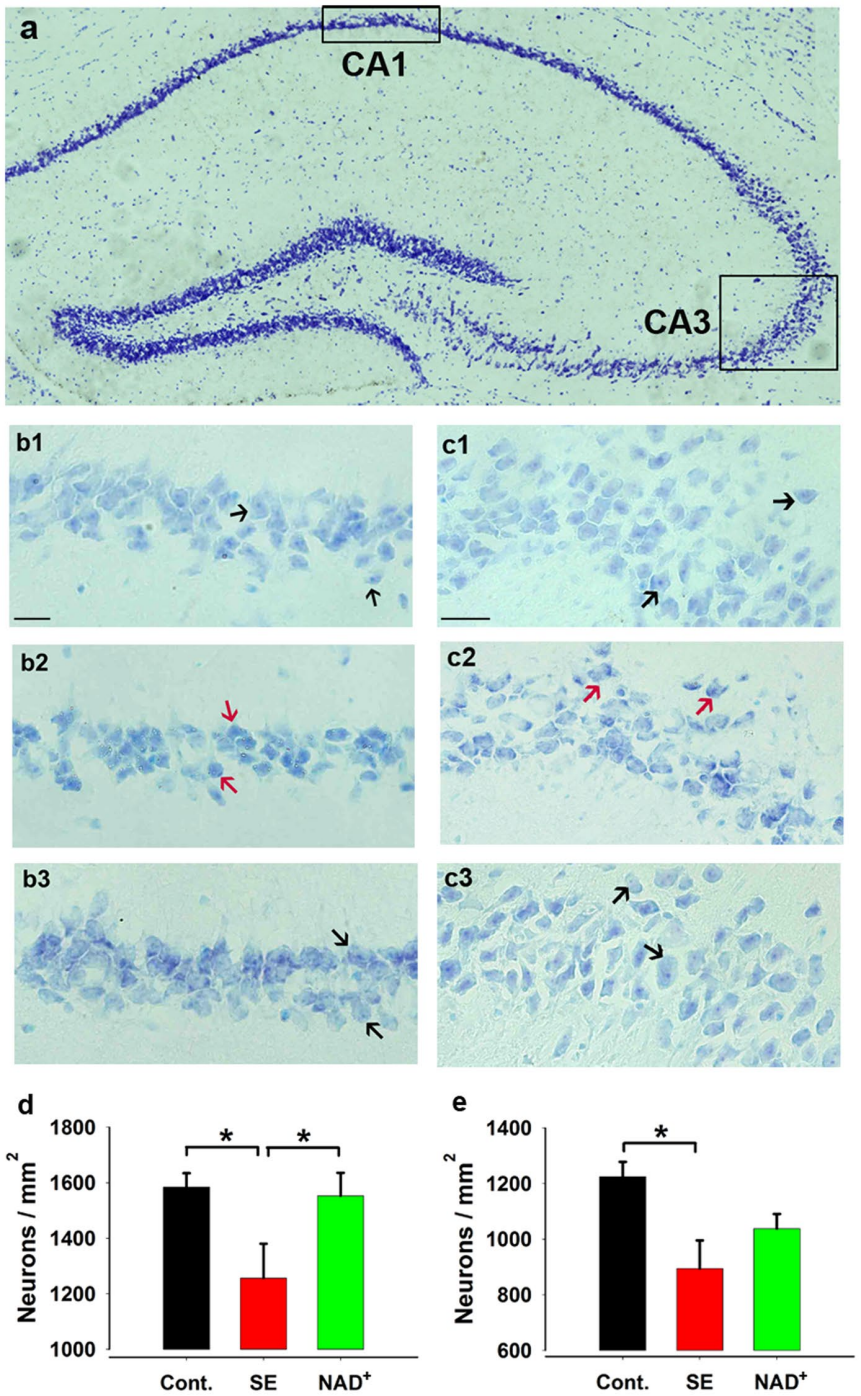
Early-stage intervention with NAD^+^ suppressed neuronal loss in hippocampus of SE model mice at SRS stage. (**a**, **b**1–3, **c**1–3) Coronal dorsal hippocampal sections were underwent Nissl staining with 0.1% thionine. (**a**) The regions used for cell counting in CA1 and CA3. Examples of intact neurons or shrunken abnormal neurons are marked with black and red arrows, respectively, in the CA1 region (**b**1–**b**3) and the CA3 region (**c**1–**c**3). Scale bars, 20 µm. Statistical results of CA1 (**d**) and CA3 (**e**). The vertical axis shows the number of intact neurons per mm^2^. Cont., SE, and NAD^+^ represent the control, SE model mice, and NAD^+^-treated SE mice, respectively. Each group has 4 mice (six sections per mice). Data are represented as mean ± SEM. *P < 0.05, **P < 0.01, ***P < 0.001.

It has been previously demonstrated that the pathogenesis of epileptogenesis involves neuronal apoptosis^5, 6^. Thus, we performed Nissl staining to confirm neuronal loss in the hippocampus of SE model mice at the SRS stage. Numerous intact normal cells were observed in the CA1 and CA3 regions of the hippocampus in the sections of control and NAD^+^-treated SE model mice. Numerous shrunken, condensed degenerating neurons were observed in the CA1 and CA3 regions of hippocampus in SE mice. We calculated the number of intact neurons in a certain area presented in Fig. 4a. Compared with control mice, the number of normal neurons significantly decreased in CA1 (Fig. 4d, control mice, 1584 ± 50, N = 4, number of sections used for Nissl staining S = 6 per mice; SE mice, 1257 ± 123, N = 4, S = 6 per mice) and in CA3 (Fig. 4e, control mice, 1224 ± 54, N = 4, S = 6 per mice; SE mice, 895 ± 101, N = 4, S = 6 per mice) (one-way ANOVA for CA1, F(2, 9) = 3.97, *P* = 0.058; LSD post hoc, SE vs control, *P* = 0.031; CA3, F(2, 9) = 5.17, *P* = 0.032; SE vs control, *P* = 0.011). NAD^+^ treatment significantly attenuated neuronal loss in CA1 (Fig. 4d, 1552 ± 83, N = 4, S = 6 per mice) (LSD post hoc, NAD^+^ vs SE, *P* = 0.046) but not in CA3 (Fig. 4e, 1038 ± 53, N = 4, S = 6 per mice) (LSD post hoc, NAD^+^ vs SE, *P* = 0.197). We also counted the number of intact normal granule cells in the DG region and found no significant differences among the three groups (one-way ANOVA, F(2,7) = 0.469, *P* = 0.644; control group, 5542 ± 581, N = 4, S = 6 per mice; SE, 5272 ± 683, N = 3, S = 6 per mice; NAD^+^, 6225 ± 823, N = 3, S = 6 per mice). Thus, our data indicated that early-stage intervention with NAD^+^ inhibited neuronal loss in the CA1 region of the hippocampus in SE model mice at the SRS stage.

Taken together, these results demonstrate that early-stage intervention with NAD^+^ inhibits epileptogenesis.

### NAD^+^ Inhibited Neuronal Apoptosis of SE Model Mice at Early-Stage Epileptogenesis

**Figure 5:**
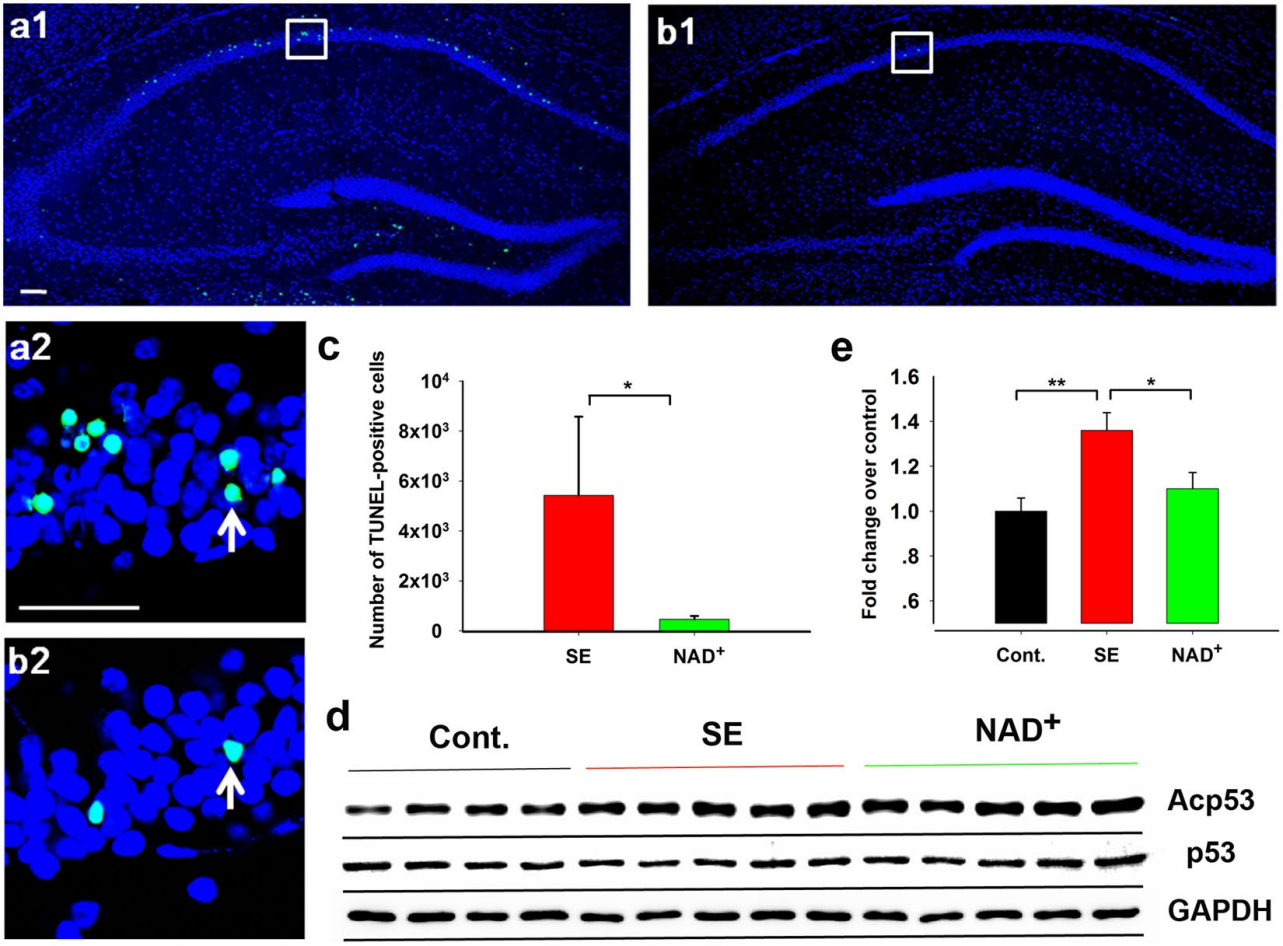
NAD^+^ injection inhibited neuronal apoptosis. (**a**2), (**b**2) Examples of apoptotic neurons in the CA1 region are marked with white arrows. (**c**) The number of double-stained cells (TUNEL-positive and DAPI-positive) of SE model mice (N = 9, S = 6 per mice) and NAD^+^-treated SE mice (N = 4, S = 6 per mice). Scale bars, 50 µm. (**d**,**e**) Western bloting showed that the Acp53/total p53 ratio was significantly increased in SE mice, and NAD^+^ treatment inhibited this increased ratio. Cont., SE, and NAD^+^ represent the control (N = 8), SE model mice (N = 9), and NAD^+^-treated SE mice (N = 10), respectively. Data are represented as mean ± SEM. *P < 0.05, **P < 0.01, ***P < 0.001.

Furthermore, to determine whether hippocampal neurons undergo apoptosis at early-stage epileptogenesis in SE model mice, and whether intervention with NAD^+^ at this stage affects neuronal apoptosis, we conducted TUNEL staining to evaluate neuronal apoptosis in the hippocampus at 72 h after SE. As shown in Fig. 5a,b, numerous TUNEL-positive signals were observed in SE mice (Fig. 5a1–2). By contrast, less, scattered TUNEL-positive signals were observed in NAD^+^-treated SE model mice (Fig. 5b1–2). We counted the number of double-stained cells (TUNEL-positive and DAPI-positive) in CA1 and calculated the total number of cells in the collected sections. We found that NAD^+^ treatment reduced the number of double-stained cells in the CA1 region in SE model mice (Fig. 5c, SE mice, 5425 ± 3150, N = 9, number of sections used for TUNEL staining S = 6 per mice; NAD^+^-treated SE mice, 468 ± 126, N = 4, S = 6 per mice; Student’s *t*-test, NAD^+^ vs SE, *P* = 0.017), confirming that early-stage intervention with NAD^+^ inhibits apoptosis of CA1 hippocampal neurons.

We also performed Western blotting to test the acetylation of p53 at lysine 120 in the hippocampus of SE model mice, because such acetylation is crucial for p53-mediated apoptosis and for transcriptional induction of proapoptotic p53 target genes^19, 20^. At 24–25 h after SE, the Acp53/p53 ratio was significantly increased in SE mice (Fig. 5d,e, one-way ANOVA. F(2, 24) = 6.525, *P* = 0.005; control mice, 1.00 ± 0.06, N = 8; SE mice, 1.36 ± 0.08, N = 9; LSD post hoc, SE vs control, *P* = 0.002). NAD^+^ treatment (1.10 ± 0.07, N = 10; LSD post hoc, NAD^+^ vs SE, *P* = 0.014) significantly reduced the Acp53/p53 ratio. These results suggest that NAD^+^ treatment inhibited pilocarpine injection-induced neuronal apoptosis through the p53-dependent pathway.

## Discussion

One of the most prominent advantages of our study is that we focused on intervention with NAD^+^ at early-stage epileptogenesis and attempted to achieve disease-modifying effects, rather than simply attenuating the seizure symptoms. In this study, NAD^+^ that was injected three times at 0.5, 12.5, and 24.5 h after SE markedly inhibited the incidence of SRS and abnormal EEG activity, reduced contextual memory impairment, and reduced neuronal loss in the CA1 region of the hippocampus in SE model mice at the chronic stage, thereby demonstrating that early-stage intervention with NAD^+^ prevents epileptogenesis. Mechanism analysis showed that exogenous NAD^+^ supply distinctly reversed the depletion of endogenous NAD^+^ and neuronal apoptosis in SE model mice at early-stage epileptogenesis, by suppressing the augmentation of Ap53/p53. All these results provide evidence that intervention with NAD^+^ prevents early-stage epileptogenesis by inhibiting p53-mediated apoptosis.

The concentration of NAD^+^ in the hippocampus of control mice obtained in our assay system was 473.8 ± 3.5 nmol/g tissue, which is consistent with the results of previous research^12^. The concentration of NAD^+^ to be injected was determined based on previous reports in other mouse models: intraperitoneal injection of 50 mg/kg NAD^+^ decreases ischemic brain damage in ischemic model mice and intraperitoneal injection of 100 mg/kg NAD^+^ alleviates doxorubicin-induced liver damage in mice^21, 22^. Based on these reports, in our experiments, we injected NAD^+^ intraperitoneally at a dose of 100 mg/kg. As shown in Fig. 1b, the concentration of NAD^+^ in the hippocampus was significantly increased in NAD^+^-treated SE mice. This finding indicated that this dose is sufficient for restoring NAD^+^ depleted in the hippocampus at, at least, 30 min after the injection in SE mice. We injected NAD^+^ three times within 24.5 h after SE onset because we found endogenous exhaustion of NAD^+^ in the hippocampus at 24.5 h after SE. This was consistent with previous study which reported the significant NAD^+^ depletion in the acquired epilepsy neuronal cell model caused by Mg^2+^-free incubation^16^. Our results demonstrated that the exogenous supplementation of NAD^+^ at 0.5, 12.5, and 24.5 h after SE can replenish NAD^+^ in the hippocampus and suppress epileptogenesis. Regarding the molecular mechanisms underlying this NAD^+^ depletion, several studies have pointed to the activation of poly(ADP-ribose) polymerase-1 (PARP-1), a NAD^+^-dependent DNA repair-associated enzyme. Conrad *et al*. found that a PARP-1 inhibitor could reverse NAD^+^ depletion in the neuronal cell or astrocytes death model^23, 24^. Moreover, it was reported that the expression of poly(ADP-ribose) (PAR; a marker of PARP-1 activity) was significantly increased in the Kainic Acid (KA)-induced epilepsy model or in the acquired epilepsy neuronal model^25, 26^.

As described in the Methods section of the previous manuscript, EEG and behaviors of mice were simultaneously recorded both in acute and chronic stages. During the acute stage, we always detected epileptic EEG activities concurrent with seizure occurrence. Supplemental Files 2 and 1 show the representative video clips for stage 4–5 seizures and the concurrent poly-spike or burst firing EEG activities, respectively. However, during the SRS stage, epileptiform discharges were rarely observed with spontaneous seizures above stage 4, as shown in Fig. 2c. Currently, we do not know exactly why we could not detect epileptiform EEG activity concurrent with spontaneous seizure occurrence, but the possible explanations are as follows: Most seizures occurring within 4–10 weeks after SE onset under our experimental conditions may be partial seizures, and our electrode, which was implanted in the area of the skull corresponding to the temporal lobe, could not detect the epileptiform EEG signals of these partial seizure. Nevertheless, as shown in Fig. 2d, statistical results of power values distinctly detected the abnormal EEG activity of SE model mice. In future experiments, it may be necessary to implant electrodes directly into the hippocampus and other brain areas to improve the EEG–video monitoring system. Representative spontaneous seizures in the SRS stage are shown in Supplemental File 3; the mice with these seizures experienced typical epileptic rearing with bilateral forelimb clonus behaviors.

According to our results, early-stage intervention with NAD^+^ inhibits epileptogenesis, which is supported by the improvement of seizure behaviours, abnormal EEG activity, contextual memory impairment, and neuronal loss of the CA1 region at the chronic SRS stage. Our result that NAD^+^ suppressed neuronal loss in the CA1 region but exerted no effect on the CA3 region may be ascribed to more serious neuronal loss in CA3 region, and the exogenous NAD^+^ supply (100 mg/kg) was not sufficient to distinctly reverse the serious neuronal loss. In addition, the vulnerability of different regions of hippocampus to pilocarpine-induced neurodegeneration is different; CA3 pyramidal cells are more sensitive than CA1, and DG granule cells are the least susceptible^27, 28^, which is consistent with our results.

In this study, we also evaluated the mechanisms underlying the effects of NAD^+^ on epileptogenesis. Numerous studies have proven that the pathogenesis of epileptogenesis involves neuronal apoptosis^5, 6^. In this study, we observed that number of TUNEL-positive neurons in CA1 region at 72 h after SE was significantly prevented by exogenous NAD^+^ injection at 0.5, 12.5, and 24.5 h after SE. In addition, the augmented Acp53/p53 ratio in SE model mice was also reversed by NAD^+^ supply. Acetylated-p53 is the active form of p53, and it regulates the expression of proapoptotic genes, such as p21 and puma, and finally induces apoptosis^19, 20, 29^. NAD^+^ may inhibit acetylation of p53 through Sirtuin1, which is an NAD^+^-dependent protein deacetylase that is highly expressed in neurons and plays a key role in apoptosis through deacetylation of p53^30, 31^. Besides, NAD^+^ may reduce oxidative stress injury-induced pathogenesis of epileptogenesis^8, 32^. Thus, additional studies are required to determine the underlying mechanisms of NAD^+^-induced prevention of epileptogenesis.

## Methods

### Animals

In this study, 5–6-week-old male C57BL/6 mice (Shanghai Laboratory Animal Center, Chinese Academy Sciences, Shanghai, China) were used for the experiments. These mice were maintained at room temperature (23 °C) with controlled illumination (12-h light and dark cycle, lights on from 12:00 a.m. to 12:00 p.m.) and ad libitum access to water and food. These mice were allowed to adapt to these conditions for at least 1 week before their use in the experiments. All experiments were conducted in accordance with the Health Guide for the Care and Use of Laboratory Animals of Shanghai Jiao Tong University. All experimental protocols were approved by the Institutional Animal Care and Use Committee of Shanghai Jiao Tong University. All efforts were made to minimise both the number and suffering of animals. A total of 172 mice were used in this study, of which 63 were evaluated using the behaviour tests (SRS video monitoring, EEG recording, fear conditioning test) and 109 were evaluated using biochemical molecular assays (Nissl staining, NAD^+^ assay, TdT-mediated dUTP nick end labelling (TUNEL) staining, and evaluation of the Acp53/p53 ratio).

### Pilocarpine-Induced SE Model

To minimise peripheral cholinergic effects, mice were administered atropine (1 mg/kg, i.p.) 30 min before being injected with pilocarpine (300 mg/kg, i.p.; Sigma, USA). In the following 60 min, seizure activity was monitored by recording behavioural videos and was graded using Racine’s scale^33^. Only mice that developed SE were used in this study. SE was defined as continuous seizures for a period longer than 30 min and was characterised as stage 4 (rearing with bilateral forelimb clonus) and stage 5 (rearing and falling, loss of postural control, and jumping). Mice that experienced SE were administered chloral hydrate (150 mg/kg, i.p.; Sangon Biotech, China) to terminate the continuous seizures. Control mice were injected with atropine and chloral hydrate (at the end of the 60-min monitoring period), as described earlier in the text, and were injected with saline instead of pilocarpine. Mice that developed SE were randomly divided into two groups, the SE group and the NAD^+^-treated SE group. Mice in the NAD^+^-treated SE group received i.p. injection of NAD^+^ (100 mg/kg, Sigma) at 30 min, 12.5 h, and 24.5 h after SE.

### EEG Recording and Video Monitoring

Three weeks after SE, three groups of mice (N = 9–10 mice per group) were surgically implanted with electrodes under stereotaxic guidance. Two 1-mm holes each for the recording and reference electrodes were drilled into the skull at the following stereotaxic coordinates from the bregma: 1.8 mm posterior and 1.4 mm left, and 1.5 mm anterior and 1.5 mm right, respectively. At least 1 week after surgery, single-channel intracranial EEG activity was acquired in freely moving mice by using the NeuroLog Electrophysiological Recording System (CED, England). The signal was amplified 1000 times, filtered from 0.1 to 500 Hz, and digitised at a sampling rate of 2000 Hz. Power spectrum analysis of 12-h EEG during the light cycle was performed offline by using Spike2 software (CED). The EEG power in each frequency band was calculated and compared among the groups. During *in vivo* EEG recording, the behaviours of mice were simultaneously video monitored using the CloudView Digital Video Recording System (Jovision Technology, China). Each mouse was continuously recorded for 24 h at a time and for a total duration of 2–3 days. The number of spontaneous seizures (stage 4 or 5, Racine’s score) and their durations and frequencies were calculated.

### Contextual Fear Conditioning Test

Seven weeks after SE, the contextual fear conditioning test was performed using a computerised fear conditioning system (Coulbourn Instruments, USA), as previously described^34^. The conditioning chamber (17.8 × 17.8 × 30.5 cm) was placed in a sound-attenuated room. On Day 1, mice were allowed to acclimatise to the chamber for 3 min. On Day 2, after being allowed to explore the chamber for 3 min, mice received three electric foot shocks (0.4 mA, 20 ms) at an interval of 30 s. On Day 3, mice were placed in the chamber again for 2 min without foot shocks, and their freezing time was assessed. We defined freezing behaviour as the absence of any movement except for breathing movement.

### Nissl Staining

Six weeks after SE, Nissl staining of 10-µm thick coronal dorsal hippocampal sections was performed using 0.1% thionine (Sangon Biotech). The number of surviving pyramidal cells in the CA1 and CA3 regions of the hippocampus within a rectangular area was counted in a blinded manner (N = 4 mice per group; six sections per mice; high magnification of 400 × ; CA1: 0.027 mm^2^, CA3: 0.048 mm^2^, DG [dentate gyrus]: 0.012 mm^2^). The following rigorous criteria were used to characterise normal surviving neurons: a centrally located nucleolus, a distinctive nucleus, and visible cytoplasm. The final results are expressed as the number of normal cells per square millimetres.

### NAD^+^ Assay

At 1, 12.5, and 24.5 h after SE (30 min after each NAD^+^ injection), the hippocampus was harvested, and the hippocampal NAD^+^ levels were measured using an enzymatic recycling assay. The tissue was homogenised with 1.5 M HCLO_4_ and kept on ice for 10 min. The homogenate was centrifuged at 12,000 × *g* for 7 min, and the supernatant was neutralised to pH 7.2 by using 3 M KOH and 1 M potassium phosphate buffer. Subsequently, the mixture was centrifuged to remove potassium perchlorate precipitation. The supernatant was mixed with a reaction buffer containing 1.7 mg methyl thiazolyl tetrazolium, 10.6 mg phenazine methosulfate, 1.3 mg alcohol dehydrogenase, 488.4 mg nicotinamide, and 2.4 mL ethanol in 36 mL Gly–Gly buffer (65 mM, pH 7.4, Sigma). The mixture was incubated at 37 °C for 7 min, and the absorbance at 556 nm was measured. The readings were calibrated with NAD^+^ standards.

### Western Blotting

Western blotting was conducted to semiquantify the levels of acetylated p53 and p53 in the hippocampus at 24–25 h after SE. The hippocampal tissues were homogenised, and the total proteins were extracted using RIPA lysis buffer (Sigma) including a protease inhibitor and a phosphorylase inhibitor (Roche, USA). The Western blots were incubated with rabbit anti-acetylated p53-K120 antibody (1:5000; Abcam, USA) and mouse anti-glyceraldehyde 3-phosphate dehydrogenase (GAPDH) antibody(1:2000; Beyotime Biotechnology, China) overnight at 4 °C. After the detection of acetylated p53 bands and GAPDH bands by using ECL detection reagents (Millipore, USA), the blots were washed in Western blotting stripping buffer (Thermo, USA) for 10 min at room temperature and incubated with anti-p53 (1:250; Santacruz, USA) for 2 h at room temperature. Western blot images were captured using ChemiDoc^TM^ Touch Imaging System (Bio-Rad). Acetylated p53 and total p53 levels were quantified through densitometry by using ImageJ software.

### *In Situ* TUNEL Staining

At 72 h after SE, mice were transcardially perfused with 4% Paraformaldehyde (PFA) (Sigma); thereafter, the brains of the mice were dehydrated and sliced coronally (30 µm). TUNEL staining of whole coronal dorsal hippocampal sections was performed using the *In Situ* Cell Death Detection Kit (Roche), according to manufacturer instructions. The sections were incubated with blocking solution (2% goat serum [Millipore] in 0.3% Triton X-100 [Sigma]) for 10 min at room temperature. The sections were incubated with TUNEL antibody (Roche) in a humidified box for 1 h at 37 °C. The nuclei were labelled with 4′,6-diamidino-2-phenylindole (DAPI) (Sigma). The brain slices were observed under a Leica confocal microscope. The number of double-stained cells (TUNEL-positive and DAPI-positive) was counted.

### Statistical Analyses

Data are presented as mean ± standard error of the mean. The results of comparisons among groups were statistically evaluated using the Chi-square test, unpaired Student’s t-test, or one-way analysis of variance (ANOVA) with the least significant difference (LSD) post hoc test, as appropriate.

### Data availability

The data that support the findings of this study are available from the corresponding authors upon request.

### Ethical publication statement

We confirm that we have read the Journal’s position on issues involved in ethical publication and affirm that this report is consistent with those guidelines.

## Electronic supplementary material

Full-length blots

Epileptic EEG activities concurrent with seizure occurrence in the acute stage

Video clips of seizures in acute stage

Video clips of spontaneous seizures in chronic stage
