# Supplementary material for: Nicotinamide adenine dinucleotide suppresses epileptogenesis at an early stage
Source: Sci Rep. 2017 Aug 4;7:7321. doi: 10.1038/s41598-017-07343-0 (PMC5544671; doi:10.1038/s41598-017-07343-0)

Nicotinamide adenine dinucleotide suppresses epileptogenesis at an early stage

Juan Liu, MSc,[1,2#] Beimeng Yang, PhD,[1#] Pei Zhou, MSc,[1#] Yingying Kong, MSc,[1] Weiwei Hu, MSc,[1] Geng Zhu, PhD,[1] Weihai Ying, PhD,[3] Weidong Li, PhD,[1*] Yun Wang, PhD,[2*] Shengtian Li, PhD,[1*]

[1] Key laboratory for the Genetics of Developmental and Neuropsychiatric Disorders (Ministry of Education), Bio-X Institutes, Shanghai Key Laboratory of Psychotic Disorders, Institute of Social Cognitive and Behavioral Sciences, and Brain Science and Technology Research Center, Shanghai Jiao Tong University, 800 Dongchuan Road, Shanghai 200240.

[2] Institutes of Brain Science, State Key Laboratory for Medical Neurobiology, Collaborative Innovation Center for Brain Science, Fudan University, Shanghai, 200032.

[3] School of Bimedical Engineering and Med-X Research Institute, Shanghai Jiao Tong University, Shanghai, 200030.

# Co-first authors

* Co-corresponding authors

Correspondence should be addressed to:

Dr. Shengtian Li, Bio-X Institutes, Shanghai Jiao Tong University, Shanghai, China.

Email: lstian@sjtu.edu.cn;

Dr. YunWang, Collaborative Innovation Center for Brain Science, Fudan University, China.

Email: yunwang@fudan.edu.cn;

Dr. Weidong Li, Bio-X Institutes, Shanghai Jiao Tong University, Shanghai, China.

Email: liwd@sjtu.edu.cn.

Full-length blots for Fig 5d from the main text

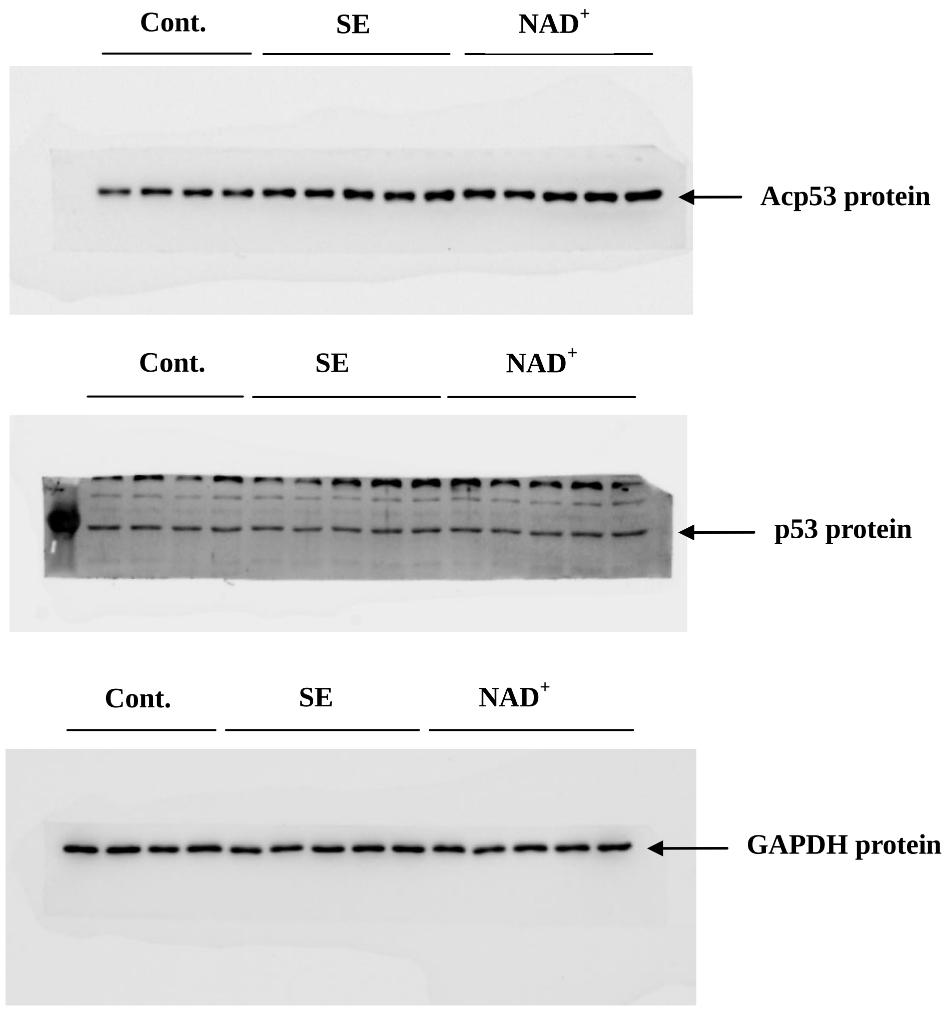

Supplement: Supplementary file 1 — Full-length blots [file 41598_2017_7343_MOESM1_ESM.pdf]
