# Supplementary material for: Nicotinamide adenine dinucleotide suppresses epileptogenesis at an early stage
Source: Sci Rep. 2017 Aug 4;7:7321. doi: 10.1038/s41598-017-07343-0 (PMC5544671; doi:10.1038/s41598-017-07343-0)

Nicotinamide adenine dinucleotide suppresses epileptogenesis at an early stage

Juan Liu, MSc,[1,2#] Beimeng Yang, PhD,[1#] Pei Zhou, MSc,[1#] Yingying Kong, MSc,[1] Weiwei Hu, MSc,[1] Geng Zhu, PhD,[1] Weihai Ying, PhD,[3] Weidong Li, PhD,[1*] Yun Wang, PhD,[2*] Shengtian Li, PhD,[1*]

[1] Key laboratory for the Genetics of Developmental and Neuropsychiatric Disorders (Ministry of Education), Bio-X Institutes, Shanghai Key Laboratory of Psychotic Disorders, Institute of Social Cognitive and Behavioral Sciences, and Brain Science and Technology Research Center, Shanghai Jiao Tong University, 800 Dongchuan Road, Shanghai 200240.

[2] Institutes of Brain Science, State Key Laboratory for Medical Neurobiology, Collaborative Innovation Center for Brain Science, Fudan University, Shanghai, 200032.

[3] School of Bimedical Engineering and Med-X Research Institute, Shanghai Jiao Tong University, Shanghai, 200030.

# Co-first authors

* Co-corresponding authors

Correspondence should be addressed to:

Dr. Shengtian Li, Bio-X Institutes, Shanghai Jiao Tong University, Shanghai, China.

Email: lstian@sjtu.edu.cn;

Dr. YunWang, Collaborative Innovation Center for Brain Science, Fudan University, China.

Email: yunwang@fudan.edu.cn;

Dr. Weidong Li, Bio-X Institutes, Shanghai Jiao Tong University, Shanghai, China.

Email: liwd@sjtu.edu.cn.

Epileptic EEG activities concurrent with seizure occurrence in the acute stage of SE.

(a) Baseline electroencephalogram (EEG) activity before pilocarpine injection. (b) Examples of poly-spike or burst firing EEG activity during the seizure (stages 4–5). This 5-s EEG span (16:16:59–16:17:04) was derived from a video clip (video#1) in the status epilepticus (SE) stage. Single-channel intracranial EEG activity was acquired in freely moving mice by using the NeuroLog Electrophysiological Recording System. The signal was amplified 1000 times, filtered from 0.1 to 500 Hz, and digitised at a sampling rate of 2000 Hz. During in vivo EEG recording, the behaviors of mice were simultaneously video monitored using the CloudView Digital Video Recording System (Jovision Technology, China).

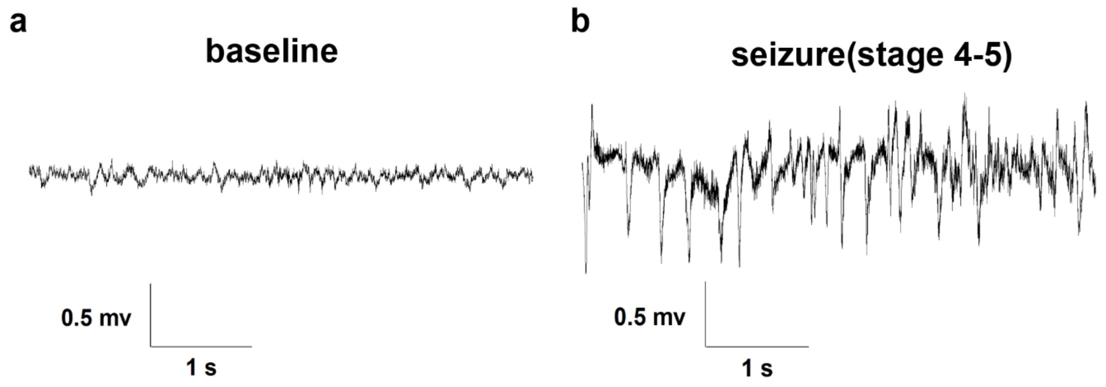

Supplement: Supplementary file 2 — Epileptic EEG activities concurrent with seizure occurrence in the acute stage [file 41598_2017_7343_MOESM2_ESM.pdf]
